# CCFormer: Cross-Modal Cross-Attention Transformer for Classification of Hyperspectral and LiDAR Data

**DOI:** 10.3390/s25185698

**Published:** 2025-09-12

**Authors:** Hufeng Guo, Baohui Tian, Wenyi Liu

**Affiliations:** 1 State Key Laboratory of Dynamic Measurement Technology, School of Instrument and Electronics, North University of China, Taiyuan 030051, China; 2 Department of Transportation Information Engineering, Henan College of Transportation, Zhengzhou 451460, China

**Keywords:** hyperspectral image, light detection and ranging, transformer, convolutional neural networks

## Abstract

The fusion of multi-source remote sensing data has emerged as a critical technical approach to enhancing the accuracy of ground object classification. The synergistic integration of hyperspectral images and light detection and ranging data can significantly improve the capability of identifying ground objects in complex environments. However, modeling the correlation between their heterogeneous features remains a key technical challenge. Conventional methods often result in feature redundancy due to simple concatenation, making it difficult to effectively exploit the complementary information across modalities. To address this issue, this paper proposes a cross-modal cross-attention Transformer network for the classification of hyperspectral images combined with light detection and ranging data. The proposed method aims to effectively integrate the complementary characteristics of hyperspectral images and light detection and ranging data. Specifically, it employs a two-level pyramid architecture to extract multi-scale features at the shallow level, thereby overcoming the redundancy limitations associated with traditional stacking-based fusion approaches. Furthermore, an innovative cross-attention mechanism is introduced within the Transformer encoder to dynamically capture the semantic correlations between the spectral features of hyperspectral images and the elevation information from light detection and ranging data. This enables effective feature alignment and enhancement through the adaptive allocation of attention weights. Extensive experiments conducted on three publicly available datasets demonstrate that the proposed method exhibits notable advantages over existing state-of-the-art approaches.

## 1. Introduction

In recent decades, remote sensing technology has witnessed remarkable advancements. Notably, imaging technology has undergone rapid development, paving the way for more in-depth analysis in the field of intelligent Earth observation. With enhanced data acquisition capabilities—including high-resolution imagery, multi-spectral and hyperspectral sensing, and synthetic aperture radar—remote sensing data are now widely applied in critical domains such as land use planning, mineral resource exploration, agricultural quality assessment, precision agriculture, ecological monitoring, and national defense [1,2,3,4]. Among these applications, pixel-based remote sensing image classification serves as a core research task and has become a key driver in advancing remote sensing technology toward greater quantification and intelligence through the accurate identification of ground objects and detailed information extraction.

In the early stages, research on remote sensing image classification primarily relied on single data sources, such as multispectral or hyperspectral imagery [5]. Currently, hyperspectral data has emerged as a prominent resource in the field of remote sensing, attracting significant attention due to its hundreds or even thousands of spectral channels, which enable the acquisition of rich spectral information. It demonstrates notable advantages in identifying surface objects and has yielded favorable application outcomes [2,4]. However, the use of hyperspectral image (HSI) data alone for land classification is constrained by two key phenomena: “same object with different spectra” (where the spectral characteristics of the same ground object vary under different environmental conditions) and “different objects with the same spectra” (where distinct ground objects exhibit similar spectral signatures). These phenomena limit classification accuracy [6]. For instance, in urban environments, sidewalks and building rooftops may be constructed from similar materials, resulting in highly similar spectral curves. Additionally, environmental factors such as illumination conditions and atmospheric scattering can alter the spectral properties of surface objects, further exacerbating the “different objects with the same spectra” issue [1]. Consequently, relying solely on spectral data makes it challenging to accurately distinguish between certain land use and surface cover types.

Light Detection and Ranging (LiDAR) data can generate precise three-dimensional profiles by measuring the distance between the sensor and the Earth’s surface, thereby providing high-precision topographic and structural information [3]. Considering the challenges in HSI classification mentioned in the previous paragraph, the elevation information provided by LiDAR offers critical vertical dimension data that can help mitigate these classification ambiguities. Therefore, the fusion of hyperspectral and LiDAR data enables the full utilization of the complementary strengths of both data modalities, thereby effectively enhancing the classification accuracy [7].

The integration of HSI and LiDAR data has garnered considerable attention within the domain of multimodal remote sensing. However, effectively combining the rich spectral information from HSI with the elevation features provided by LiDAR remains a critical challenge. Traditional classification approaches predominantly emphasize data-level fusion strategies [8]. For instance, Ghamisi et al. [9] employed the Attribute Profiles model to capture spatial characteristics of both data types and further extracted multimodal spatial features using the Extinction Profiles technique. Although conventional machine learning techniques—such as Support Vector Machine (SVM) [10], Extreme Learning Machine (ELM) [11], and Random Forest (RF) [12]—have demonstrated moderate success in the classification of multimodal remote sensing data, these shallow models are limited in their ability to uncover deep and complex data relationships. Particularly when confronted with the nonlinear characteristics of HSI data, such methods often compromise the original spatial and spectral structure, leading to the loss of valuable implicit information [7]. For example, SVM and RF rely heavily on manually engineered features and linear assumptions, which hinder their capacity to accurately model the intricate nonlinear relationships inherent in high-dimensional hyperspectral data [13]. Furthermore, these models frequently encounter the “curse of dimensionality”—even with sufficient training samples, their classification performance falls short compared to that of deep learning approaches [14].

Compared with traditional approaches, deep learning methods demonstrate superior feature representation capabilities, enabling the automatic extraction of multi-level and highly abstract features from raw data [15,16]. Among these, Convolutional Neural Networks (CNNs) are widely employed as fundamental models owing to their effective local receptive fields and parameter sharing mechanisms [17]. Zhao et al. [18] proposed a dual-interactive hierarchical adaptive fusion network built upon a dual-branch CNN architecture. This network is capable of extracting discriminative, high-level semantic features from both HSI and LiDAR data, thereby achieving improved classification performance. Huang et al. [19] proposed a method based on convolutional neural networks. By incorporating a cross-attention mechanism, significant spatial weights are assigned to LiDAR data with respect to HSI, thereby enhancing the interaction between the two modalities and fully exploiting the complementary information from data fusion. Ge et al. [20] proposed a multi-scale CNN framework that integrates parameter sharing with a local-global cross-attention mechanism. This approach enables the joint deep semantic representation and data fusion of HSI and LiDAR data. Liu et al. [21] developed a multi-scale spatial feature module and achieved feature fusion through concatenation operations, thereby proposing a multi-scale and multi-directional feature extraction network. Indeed, the design of multi-scale modules and the incorporation of attention mechanisms into CNNs can mitigate the limitation of fixed receptive fields and enhance the capability of extracting remote sensing features. However, stacking such modules significantly increases the number of parameters and computational complexity [22,23]. In addition, due to the inherent local receptive field of convolutional operations, these models still face challenges in effectively capturing long-range dependencies across different scenarios and in handling the long-sequence characteristics of spectral features.

Given the exceptional capability of Vision Transformers (ViT) in modeling long-distance dependencies in visual tasks, researchers have incorporated the Transformer architecture into remote sensing image classification tasks [24,25]. Yang et al. [26] developed a stackable modal fusion block as the central component of the model and introduced a multi-modal data fusion framework tailored for the integration and classification of HSI and LiDAR data, achieving an overall classification accuracy of 99.91%. Huang et al. [27] integrated a CNN with the latest Transformer architecture and proposed a novel multi-modal cross-layer fusion Transformer network, aiming to enhance both the stability and performance of the model. Sun et al. [28] proposed a multi-scale 3D-2D hybrid CNN feature extraction framework combined with a lightweight attention-free fusion network for multi-source data, based on the integration of a convolutional neural network and Transformer architectures, thereby substantially enhancing the performance of joint classification. Ni et al. [29] focused on the selective convolutional kernel mechanism and the spectral-spatial interaction transformer for feature learning, and subsequently proposed a selective spectral-spatial aggregation transformer network. Roy et al. [30] introduced cross-attention, extended the traditional self-attention mechanism, and proposed a novel multi-modal deep learning framework that effectively integrates remote sensing data.

Although current methodologies have demonstrated substantial improvements in classification accuracy, the integration of remote sensing data for fusion-based classification continues to present certain challenges.

1. Feature extraction remains suboptimal. Solely relying on a simplistic network architecture to extract basic information from HSI and LiDAR data limits the in-depth exploration of the complementary characteristics of multimodal data. For instance, the integrated representation and cross-scale interaction among spectral, spatial, and elevation features are not effectively achieved, leading to underutilization of feature diversity.
2. There exists a feature fusion defect. Relying solely on simple feature stacking and fusion overlooks the correlations between heterogeneous features, which in turn limits the model’s final decision-making capability.

To address the aforementioned challenges, this paper proposes a cross-modal cross-attention Transformer (CCFormer) network that enables efficient fusion and classification through multi-scale feature interaction and semantic guidance. The network features a three-level architectural design. First, shallow feature extraction is performed using a dual-stream pyramid module. In the spectral branch, multi-scale convolutional kernels are employed to capture fine-grained spectral features from hyperspectral data, while the LiDAR branch utilizes multi-scale convolutional kernels to extract elevation structure features. Second, a cross-attention Transformer encoder is introduced to achieve cross-modal feature alignment and bidirectional semantic guidance through a cross self-attention mechanism. Finally, class probabilities are generated via a lightweight classification head. The proposed framework effectively mitigates the redundancy caused by simple feature concatenation and enhances the model’s decision-making capability through pyramid-based multi-scale feature refinement and cross-modal attention interaction.

The main contributions of this work are summarized as follows.

1. In the shallow feature extraction stage, considering the rich spectral characteristics of HSI and the elevation information provided by LiDAR, a pyramid spectral multi-scale module and a spatial multi-scale module are, respectively, designed. Through a two-level multi-scale feature extraction process, the features are progressively refined, allowing for a comprehensive exploration of the intrinsic representations of each modality. This lays a solid foundation for subsequent cross-attention fusion by providing complementary features.
2. A CCFormer is proposed to enable bidirectional interaction between HSI and LiDAR features by reformulating the attention calculation paradigm. Utilize the spectral features of HSI as the query, and the elevation-structure features of LiDAR as the key and value. Dynamically assign weights to enhance the correlation between heterogeneous features. Meanwhile, employ the LiDAR features as the query and the HSI features as the key and value to construct a bidirectional attention mechanism.
3. Performance evaluation was conducted on three representative remote sensing datasets. The experimental results demonstrate that the proposed algorithm surpasses the current state-of-the-art methods.

## 2. Related Work

### 2.1. Multiscale Feature Extraction

In image segmentation and classification tasks, multi-scale methods enhance model performance by capturing features across various spatial dimensions [31]. The theoretical foundation of these methods can be traced back to the concept of scale space introduced in 1962, with a significant quantitative advancement achieved in 1983 through the application of Gaussian filters. Initially, multi-scale features were primarily extracted using image pyramids. However, this approach has two major limitations. First, it lacks the capability for effective cross-scale feature interaction and analysis, making it difficult to establish meaningful associations across different scales. Second, the Gaussian function, which forms the core of this method, tends to blur edge and detail information in the image, thereby reducing the accuracy in locating target boundaries [14]. With the advent of deep learning, architectures such as GoogLeNet (featuring the Inception module) and VGGNet in 2014 advanced multi-scale modeling capabilities [32,33]. In recent years, innovations such as the Feature Pyramid Network (FPN) and skip connections have been introduced [34]. More recently, hybrid architectures combining CNNs and Transformers have enabled more sophisticated feature fusion, leading to notable improvements in both classification accuracy and the precision of segmentation boundary detection [25].

Owing to the high-dimensional nature of the data and the complex spectral characteristics inherent in HSIs, multi-scale approaches have emerged as a crucial strategy for effective feature extraction [24,25,26,27,28]. Meanwhile, the application of multi-scale methods in the field of remote sensing has also become a research hotspot.

### 2.2. Vision Transformer

Since its introduction in 2017, the Transformer model has overcome the limitations of traditional recurrent neural network (RNN) architectures by employing a self-attention mechanism. This innovation enables the efficient capture of long-range dependencies and supports parallel computation, thereby driving significant advancements in the field of natural language processing (NLP) [35]. The core self-attention mechanism enhances semantic modeling by dynamically assigning weights to features within the input sequence, establishing the Transformer as the dominant architecture for tasks such as machine translation. In 2020, ViT extended the pure Transformer framework into the domain of computer vision [36]. By utilizing patch-based image representation and hierarchical attention mechanisms, ViT achieved performance on image classification tasks that was comparable to or exceeded that of state-of-the-art CNNs, demonstrating the potential for cross-modal generalization. This milestone not only broadens the applicability of the Transformer architecture but also highlights the universal strengths of self-attention in modeling global feature relationships, signifying a paradigm shift in deep learning from localized convolutional operations to global, dynamic feature interactions.

**Figure 1:**
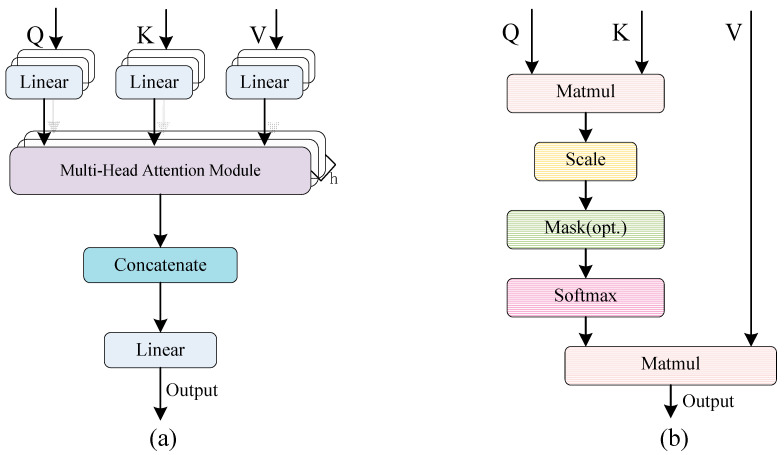
The multi-head self-attention mechanism within the Transformer encoder: (**a**) Multi-Head Attention; (**b**) Scaled Dot-Product Attention mechanism.

As illustrated in Figure 1, the multi-head self-attention mechanism, which serves as a core component of the Transformer encoder, enables efficient global feature interaction by performing parallel computations across multiple scaled dot-product attention modules. We define a sequence of n entity vectors $\left\{x_{1},x_{2},\cdot \cdot \cdot ,x_{n}\right\}$ as the input matrix $X\in R^{n\times d}$, where d denotes the embedding dimension of each entity. The purpose of the self-attention mechanism is to encode each entity using global contextual information, thereby capturing the interactions among all n entities.

In the specific implementation, through three learnable weight matrices $W^{Q}\in R^{d\times d_{q}}$, $W^{K}\in R^{d\times d_{k}}$, and $W^{V}\in R^{d\times d_{v}}$ (where the dimensions of the query and the key are the same, i.e., $d_{q}=d_{k}$), the input X is linearly projected, respectively, to obtain the query matrix Q, the key matrix K, and the value matrix V. This process can be expressed by the following formula:

(1) $$Q=XW^{Q},\;K=XW^{K},\;V=XW^{V}$$

Subsequently, compute the scaled dot-product attention $\alpha_{ij}$ between the query vector $q_{i}$ (derived from matrix Q) and the key vector $k_{j}$ (obtained from K), This process can be represented by the following formula:

(2) $$\alpha_{ij}=soft\max\left(\frac{q_{i}\cdot k_{j}^{T}}{\sqrt{d_{k}}}\right)=\frac{\exp(q_{i}\cdot k_{j}^{T}/\sqrt{d_{k}})}{\sum_{j}\exp(q_{i}\cdot k_{j}^{T}/\sqrt{d_{k}})}$$

Among them, the term $\sqrt{d_{k}}$ denotes the scaling factor, which corresponds to the dimension of $q_{i}$ and $k_{j}$. Then, applying the derived attention map α (weight parameter matrix, consisting of the elements denoted by $\alpha_{ij}$) to the value matrix V generates the attention output, which can be represented by the following formula:

(3) $$A=\sum_{i}\sum_{j}\alpha_{ij}\cdot v_{j}$$

where A is the output of single-head attention, obtained by computing a weighted sum based on the attention scores. $v_{j}$ is the *j*-th column vector of matrix V.

The multi-head self-attention mechanism partitions the input features into h distinct sub-spaces. Each attention head independently computes the attention scores, after which the results are concatenated and integrated. This mechanism enables multi-dimensional parallel modeling, there by simultaneously improving feature representation and enhancing the capability to capture complex patterns. The corresponding representation formula is as follows:

(4) $$MH=Concat\left(A_{1},A_{2},\ldots ,A_{h}\right)W$$

where $MH\in R^{h\times n}$ represents the multi-head attention of h heads, and $W\in R^{hd_{v}\times d}$ represents the learnable weight matrix. $Concat\left(\cdot \right)$ denote the Concatenate functions.

## 3. Methodology

In this section, the overall architecture of the proposed method is introduced, followed by a detailed analysis of its key internal modules, which include shallow feature extraction module and a cross-attention Transformer.

### 3.1. Overall Architecture

**Figure 2:**
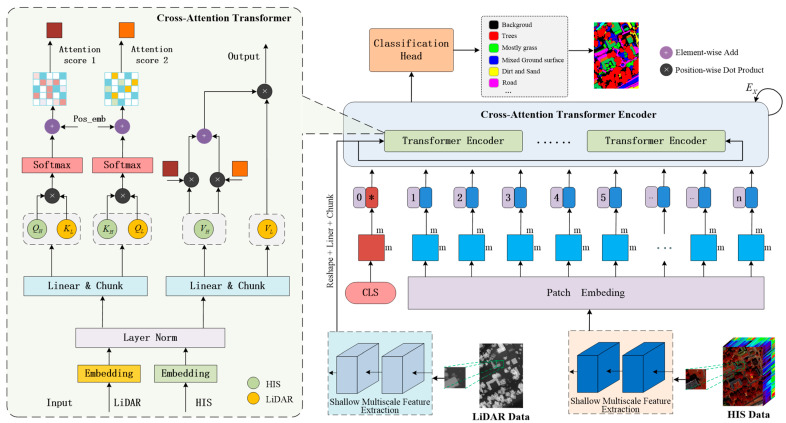
Overview framework of the proposed CAFormer-based HSI-LiDAR joint classification network. In the figure, the tokens marked with an asterisk (*) denote classification tokens.

Considering the complementary characteristics of HSIs—which offer high spectral resolution but limited spatial detail—and LiDAR data—which provide rich three-dimensional structural information but lack spectral features—this study addresses the challenge of enhancing the robustness of ground object classification in scenarios with limited training samples. To this end, a multi-scale cross-guided CCFormer framework is proposed. By leveraging a cross-modal feature mutual guidance and fusion mechanism, the framework effectively integrates the spectral-spatial information from hyperspectral images with the elevation-structural information from LiDAR data, thereby mitigating classification ambiguities caused by the limitations of single-source data. Figure 2 illustrates the multi-modal cross-guidance and fusion strategy employed in the proposed framework. This framework achieves collaborative enhancement of spectral details and geometric features through a dual-guided cross-attention mechanism, offering a novel solution for small-sample classification in complex scenarios. The method follows a hierarchical architecture, comprising a shallow multi-scale feature extraction module, a CCFormer encoder, and a classification decision layer, thereby establishing an end-to-end cross-modal fusion classification framework.

The inputs to the model consist of the raw HSI data and LiAR data, denoted as $X_{H}\in R^{W\times H\times L}$ and $X_{L}\in R^{W\times H}$, respectively, where W and H correspond to the spatial width and height dimensions of the data, while L denotes the spectral dimension of the HSI. During the data preprocessing phase, a square window of size s×s is applied in a sliding manner pixel by pixel, and each resulting s×s block is treated as an individual sample. The samples derived from the HSI data and LiDAR data are denoted as $X_{h}\in R^{s\times s\times L}$ and $X_{l}\in R^{s\times s}$, respectively. Subsequently, a shallow multiscale feature extraction process is applied to each sample, involving a two-level multiscale feature mining strategy. With respect to the spectral dimension of HSI, multiscale band grouping is performed along with local spectral correlation modeling. Regarding the spatial dimension of LiDAR data, multiscale spatial filtering and local geometric structure analysis are implemented. In the cross-modal feature fusion stage, the HSI features and LiDAR features are fed into the cross-attention Transformer encoder unit. Bidirectional information exchange is facilitated through cross-modal feature interaction guidance, enabling comprehensive feature fusion at multiple levels via a multi-level attention mechanism. Finally, the classification head performs the final classification by mapping the extracted high-dimensional features into the corresponding category label space.

It is worth noting that traditional HSIC methods typically flatten either a single-pixel spectral vector or the spectral information of a local region into a one-dimensional vector to serve as input tokens for the Transformer. In contrast, this study employs a per-band feature serialization strategy. Specifically, given an input feature $X_{h}\in R^{m\times m\times b}$, a linear projection is applied to generate b band-level tokens, as shown in the following equation:

(5) $$X_{tokens}=\left[x_{seq}^{1};x_{seq}^{2};\ldots ;x_{seq}^{b}\right],\left(x_{seq}^{i}\in R^{m\times m\times 1}\right)$$

where m×m represents the spatial dimension, b represents the spectral dimension, $X_{tokens}$ is a sequence of tokens (a total of b tokens), and $x_{seq}^{i}$ is a single token. This approach aims to provide a structured input format that facilitates the evaluation of the importance of each spectral band, particularly when integrated with LiDAR data in subsequent analyses.

### 3.2. Shallow Multiscale Feature Extraction

In the shallow feature extraction stage, considering the distinct characteristics of HSI and LiDAR data, a spectral pyramid-based multiscale feature extraction module and a spatial pyramid-based multiscale feature extraction module are designed, respectively. The former achieves multi-granularity decoupling of spectral features through multi-scale band grouping and local spectral correlation modeling. The latter enables multi-level decomposition of spatial features by employing multi-scale spatial filtering and local geometric structure analysis.

**Figure 3:**
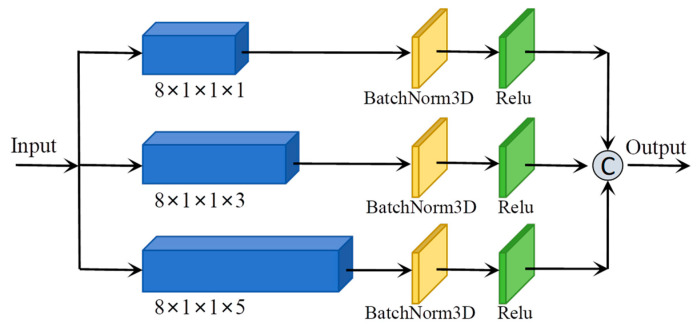
Spectral Pyramid-Based Multiscale Feature Extraction Module.

As illustrated in Figure 3, the Spectral Pyramid-Based Multiscale Feature Extraction Module comprises a spectral-dimensional pyramid structure composed of three layers. The pyramid structure performs feature extraction at multiple scales by utilizing convolutional kernels of dimensions $\left(1\times 1\times 1\right)$, $\left(1\times 1\times 3\right)$, and $\left(1\times 1\times 5\right)$ respectively along the spectral dimension. Following convolution in each layer, batch normalization is applied to standardize the data distribution across batches, thereby maintaining a relatively stable input distribution for subsequent layers. Subsequently, the ReLU activation function is employed to introduce nonlinearity, which facilitates faster network convergence and helps mitigate the risk of overfitting. This structure can be mathematically represented by the following equation:

(6) $$F_{h1}\left(X_{h}\right)=\partial \left(\beta \left(3DC\mathrm{on}v_{1\times 1\times 1}(X_{h})\right)\right)$$

(7) $$F_{h2}\left(X_{h}\right)=\partial \left(\beta \left(3DC\mathrm{on}v_{1\times 1\times 3}(X_{h})\right)\right)$$

(8) $$F_{h3}\left(X_{h}\right)=\partial \left(\beta \left(3DC\mathrm{on}v_{1\times 1\times 5}(X_{h})\right)\right)$$

(9) $$F_{hout}=Cat\left\{F_{h1}\left(X_{h}\right),F_{h2}\left(X_{h}\right),\left.F_{h3}\left(X_{h}\right)\right\}\right.$$

where $X_{h}\in R^{s\times s\times L}$ represents a sample of the input HSI, $F_{hi}$ and $F_{hout}$ denote the functional output of the i-th layer of the pyramid and the overall output features of the pyramid, respectively. Additionally, $\partial \left(\cdot \right)$, $\beta \left(\cdot \right)$, $3DConv\left(\cdot \right)$ and $Cat\left(\cdot \right)$ denote the ReLu, 3DBatchNorm, 3Dconvolution, and Concatenate functions, respectively.

**Figure 4:**
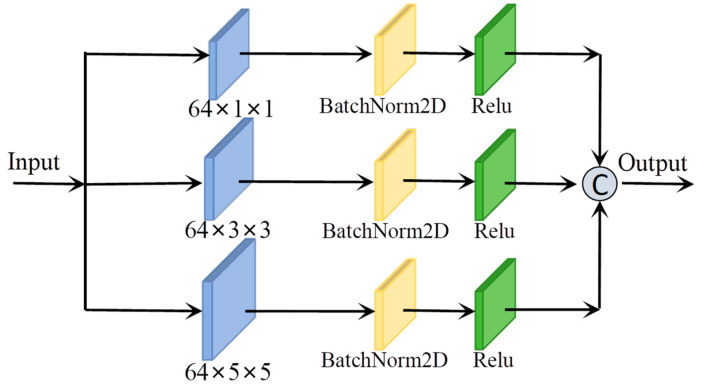
Spatial Pyramid-Based Multiscale Feature Extraction Module.

As illustrated in Figure 4, the Spatial Pyramid-Based Multiscale Feature Extraction Module comprises a spatial dimension pyramid formed by three layers of convolutional kernels with kernel sizes of $\left(1\times 1\right)$, $\left(3\times 3\right)$, and $\left(5\times 5\right)$, respectively. Its structure is similar to that of the Spectral Pyramid-Based Multiscale Feature Extraction Module. The difference is that one is based on two dimensions and the other is based on three dimensions. The module can be mathematically represented by the following equation:

(10) $$F_{l1}\left(X_{l}\right)=\partial \left(\beta \left(2DC\mathrm{on}v_{1\times 1}(X_{l})\right)\right)$$

(11) $$F_{l2}\left(X_{l}\right)=\partial \left(\beta \left(2DC\mathrm{on}v_{3\times 3}(X_{l})\right)\right)$$

(12) $$F_{l3}\left(X_{l}\right)=\partial \left(\beta \left(2DC\mathrm{on}v_{5\times 5}(X_{l})\right)\right)$$

(13) $$F_{lout}=Cat\left\{F_{l1}\left(X_{l}\right),F_{l2}\left(X_{l}\right),\left.F_{l3}\left(X_{l}\right)\right\}\right.$$

where $X_{l}\in R^{s\times s}$ represents a sample of the input HSI, $F_{li}$ and $F_{lout}$ denote the functional output of the i-th layer of the pyramid and the overall output features of the pyramid, respectively, and $2DConv\left(\cdot \right)$ represents the 2Dconvolution function.

The two feature extraction modules have similar structures. The primary distinction lies in their operational spaces: one performs multi-scale feature extraction in the planar domain, with a focus on spatial feature extraction; the other operates in the three-dimensional space, emphasizing spectral feature extraction.

### 3.3. Cross-Attention Transformer

As the core component of the Transformer architecture, the multi-head self-attention mechanism plays a crucial role in capturing long-range dependencies among features. Inspired by the cross-modal complementarity between elevation features and spectral-spatial features in remote sensing scenarios, this study enhances the multi-head self-attention mechanism by incorporating a feature interaction strategy, thereby constructing a cross-attention Transformer module. The architectural design of CCFormer is elaborated in the left portion of Figure 2.

The query vector matrix Q, as a central element of the self-attention mechanism, functions as a dynamic interface that facilitates cross-modal feature interaction. To effectively integrate multi-source remote sensing data, this mechanism computes similarity scores between the query vector matrix Q and the key vector matrix K across different modalities, followed by a weighted aggregation of the value vector matrix V, thereby enhancing the spatial-spectral correlation within key regions. Specifically, the query vector matrix $Q_{L}$ from the LiDAR branch offers attention-based guidance for the spectral features of the HSI, whereas the query vector matrix $Q_{H}$ from the HSI branch assesses the discriminative significance of the LiDAR elevation data, establishing a bidirectional cross-modal attention framework. This interactive guidance approach enables the adaptive fusion of elevation and spectral-spatial information through dynamic feature weighting, laying a theoretical foundation for the collaborative representation of multi-modal remote sensing data.

Specifically, the token sequences of HSI and LiDAR are first subjected to feature normalization using LayerNorm. Subsequently, each modality undergoes feature projection through linear transformation and chunking operations to generate the corresponding query matrix ($Q_{H}$ and $Q_{L}$), key matrix ($K_{H}$ and $K_{L}$), and value matrix ($V_{H}$ and $V_{L}$). In the cross-modal interaction phase, the affinity score is computed by constructing the similarity matrix between the query vector from one modality and the key vector from the other. To preserve spatial relative position information, rotary position encoding is integrated into this computation. Finally, regularization is applied through the Dropout mechanism. This process can be represented by the following formula:

(14) $$Att_{1}=Dp\left(soft\max\left(\frac{q_{l}\cdot k_{h}^{T}}{\sqrt{d}}\right)+Pos\left(q_{l},k_{h}\right)\right)$$

(15) $$Att_{2}=Dp\left(soft\max\left(\frac{q_{h}\cdot k_{l}^{T}}{\sqrt{d}}\right)+Pos\left(q_{h},k_{l}\right)\right)$$

where $Att_{1}$ and $Att_{2}$ denote the similarity scores between the LiDAR elevation feature query vector $q_{l}$ and the HSI key vector $k_{h}$, and between the HSI query vector $q_{h}$ and the LiDAR key vector $k_{l}$, respectively. $q_{h}$, $q_{l}$, $q_{l}$, and $k_{l}$ are derived from the matrices $Q_{H}$, $Q_{L}$, $K_{H}$, and $K_{L}$, respectively. The function $Pos\left(\cdot \right)$ refers to the rotary position embedding operation, and $Dp\left(\cdot \right)$ denotes the dropout regularization operation.

After that, perform a weighted sum of the two sets of cross-modal attention scores $Att_{1}$ and $Att_{2}$ with a set of value vectors $v_{h}$, respectively. The resulting output is then multiplied by another set of value vectors $v_{l}$ to derive the final single-head attention output Att. As illustrated in the following equation:

(16) $$Att=Lin\left(\left(\left(Att_{1}\cdot v_{h}\right)+\left(Att_{2}\cdot v_{h}\right)\right)\cdot v_{l}\right)$$

where $v_{h}$, and $v_{l}$ are derived from the matrices $V_{H}$ and $V_{L}$, respectively. $Lin\left(\cdot \right)$ represents a linear function, specifically expressed as $Y=XW^{l}+b$ ($W^{l}$ denotes the weight matrix and b represents the bias term, both parameters are learnable). Subsequently, the output of multi-head attention is obtained through a weighted concatenation of the outputs from individual single-head attention mechanisms. The process is illustrated in Equation (4) presented above.

Then, the output of the multi-head attention is first combined with the original input through a residual connection, which helps alleviate the issue of gradient vanishing. Following this, a feed-forward neural network is applied to extract high-order features, ultimately yielding the output tensor $T_{out}$. This procedure can be concisely expressed using the following mathematical formulation:

(17) $$T_{out}=LN\left(LN\left(X+\mathrm{MH}\left(X\right)\right)+FFN\left(LN\left(X+\mathrm{MH}\left(X\right)\right)\right)\right)$$

where X represents the input of the multi-head attention. $MH\left(\cdot \right)$, $LN\left(\cdot \right)$ and $FFN\left(\cdot \right)$ denote the multi-head attention, the layer normalization operation, and the feed-forward neural network, respectively. And FFNx=ReLU(xW1+b1)W2+b2, $W_{1}$ and $W_{2}$ are, respectively, the weight matrix of the first layer and the weight parameter of the second layer. Both $b_{1}$ and $b_{2}$ are biases.

## 4. Experiments and Analysis

In this section, we first present the experimental data and subsequently provide a detailed description of the experimental settings. To evaluate the performance of the proposed network, we conducted comparative experiments with several widely adopted models. Furthermore, ablation studies were carried out to validate the contribution of each individual component within the model.

### 4.1. Datasets Description

To comprehensively evaluate the applicability and effectiveness of the proposed method across diverse scenarios, we selected three widely recognized remote sensing datasets: MUUFL, Trento, and Houston2013. These datasets encompass a range of typical land cover types, including parks, agricultural areas, university campuses, and urban environments. They vary in terms of spatial resolution and spectral bands, thereby enabling the simulation of realistic application conditions. Detailed parameter specifications for each dataset are provided in Table 1.

**Figure 5:**
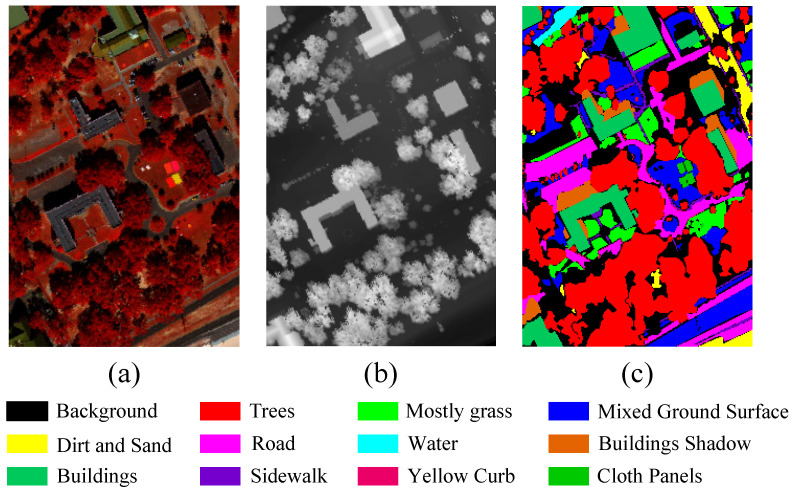
The MUUFL dataset. (**a**) Pseudocolor Representation of an HSI. (**b**) DSM of LiDAR data. (**c**) Ground-truth map.

MUUFL: The MUUFL dataset integrates HSI and LiDAR data, which were collected in November 2010 over the campus of the University of Southern Mississippi Gulf Park in Long Beach, Mississippi, USA. The hyperspectral data were acquired using the ITRES CASI-1500 sensor, covering a spectral range of 375–1050 nm (0.38–1.05 µm) with 64 spectral bands. The spatial dimensions of the HSI data are 325 × 220 pixels, with a spatial resolution of 0.54 × 1.0 m^2^. The LiDAR data were collected using the Gemini airborne system, with a spatial resolution of 0.60 × 0.78 m^2^. The dataset comprises 11 land cover classes and includes a total of 53,687 labeled samples. Figure 5 presents the false-color composite of the hyperspectral data, the digital surface model (DSM) derived from LiDAR data, and the land cover map. The distribution of samples across the training and test sets is summarized in Table 2.

**Figure 6:**
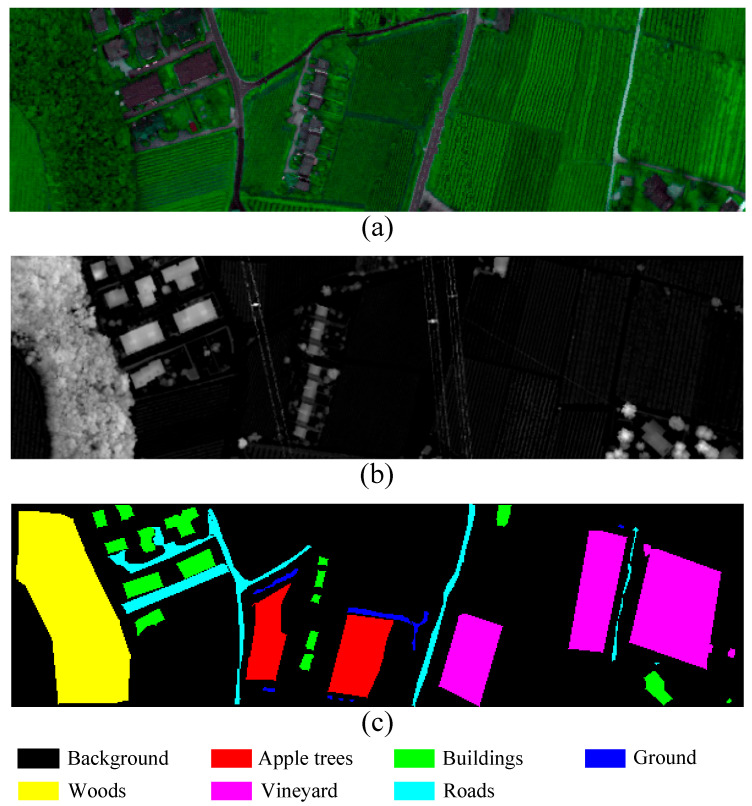
The Trento dataset. (**a**) Pseudocolor Representation of an HSI. (**b**) DSM of LiDAR data. (**c**) Ground-truth map.

Trento: The Trento dataset combines HSI and LiDAR data collected from rural areas of Trento, located in southern Italy. The HSI data was captured using the airborne Eagle sensor and comprises 63 spectral bands ranging from 0.42 to 0.99 µm. The dataset covers an area of 166 × 600 pixels with a spatial resolution of 1 m. The LiDAR data were obtained from the Optech ALTM 3100 EA airborne sensor, which produced a single raster dataset and, together with the HS data, was used to generate a DSM. This dataset is primarily intended for land cover classification involving six distinct categories and includes a total of 30,214 labeled samples. Figure 6 presents the hyperspectral false-color image of the Trento data, the LiDAR-derived DSM, and the ground truth map. The distribution of samples in the training and test sets is summarized in Table 3.

**Figure 7:**
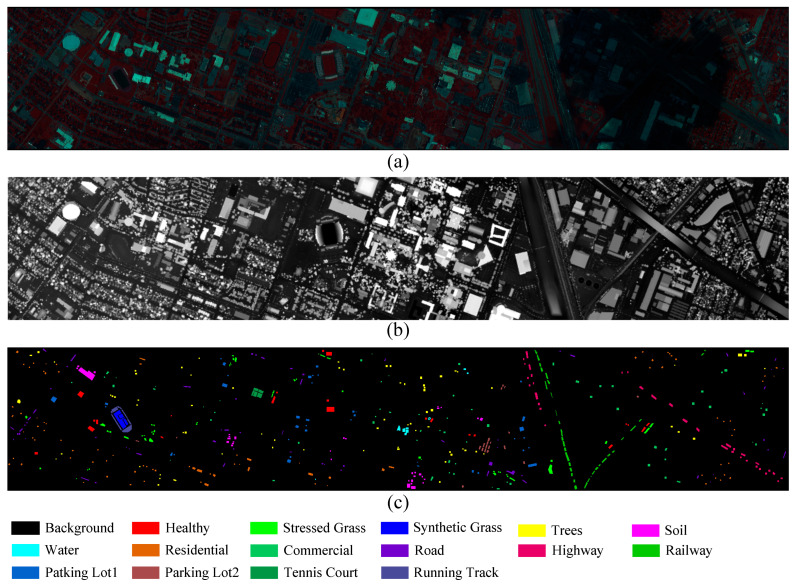
The Houston2013 dataset. (**a**) Pseudocolor Representation of an HSI. (**b**) DSM of LiDAR data. (**c**) Ground-truth map.

Houston 2013: The Houston 2013 dataset was provided by the IEEE GRSS Data Fusion Contest. It was collected in June 2012 by the National Center for Airborne Laser Mapping (NCALM) in the United States, covering areas within and around the campus of the University of Houston, Texas. This dataset combines HSI data with a DSM derived from LiDAR. The HS data was captured using the ITRES CASI-1500 sensor, comprising 144 spectral bands within the wavelength range of 0.38 to 1.05 µm. It has a spatial resolution of 2.5 m and a scene size of 349 × 1905 pixels. The LiDAR data is single-band and matches the HS data in both spatial resolution and scene dimensions. The primary objective of this dataset is to enable the classification of 15 distinct land use/land cover categories, and it includes a total of 15,029 labeled samples. Figure 7 presents the false-color HSI of the Trento data, the LiDAR-derived DSM, and the ground truth map. The distribution of samples in the training and test sets is summarized in Table 4.

### 4.2. Experimental Setup

This study constructs an experimental system using the PyTorch 1.13.1 framework. The hardware environment is equipped with an NVIDIA RTX 4090 GPU (24 GB VRAM, Santa Clara, CA, USA), and the programming environment is established on Python 3.8. The experimental parameters are configured as follows: 20 training samples are assigned to each class, with an input window size of 11 × 11 pixels. During training, the model is trained for 200 epochs, with an initial learning rate of 0.001, a batch size of 512, and the Adam optimizer is employed for model optimization; additionally, a dropout probability of 0.4 is applied to mitigate overfitting. At the network architecture level, the number of hidden channels is set to 32, the encoder adopts a two-layer structure with 6 attention heads, the temperature parameter is set to 1.0, and the hierarchical loss coefficient is set to 0.005.

The experiment uses the Overall Accuracy (OA), Average Accuracy (AA), and Kappa coefficient as quantitative indicators to evaluate the performance of the method. Among them, OA is the proportion of correctly classified pixels to the total number of pixels; AA is the average of the accuracies of each category; the Kappa coefficient measures the overall performance of the classifier by statistically analyzing the consistency between the model predictions and the true labels. The specific mathematical representation formulas are as follows.

(18) $$OA=\frac{\sum_{i=1}^{c}m_{ii}}{N_{total}}$$

(19) $$AA=\frac{1}{c}\sum_{i=1}^{c}\frac{m_{ii}}{\sum_{j=1}^{c}m_{ij}}$$

(20) $$Kappa=\frac{OA-\sum_{i=1}^{c}\left(\frac{R_{i}}{N_{total}}\cdot \frac{C_{i}}{N_{total}}\right)}{1-\sum_{i=1}^{c}\left(\frac{R_{i}}{N_{total}}\cdot \frac{C_{i}}{N_{total}}\right)}$$

Among them, $m_{ij}$ denotes the number of pixels that truly belong to class *i* but are classified as class *j* (with $m_{ii}$ representing the number of correctly classified samples). c refers to the total number of object class categories, and $N_{total}$ indicates the total number of test samples. Furthermore, $R_{i}$ and $C_{i}$ denote the sum of the *i*-th row and the sum of the *i*-th column of the confusion matrix, respectively.

### 4.3. Classification Results

To evaluate the performance of the proposed method, we conducted comparative experiments involving seven SOTA models: HybridSN [15], SSFTT [16], DCTN [23], MS2CANet [24], MFT [8], MADNet [25], and PyionNet [3]. In the experiment, HybridSN, SSFTT, and DCTN—recognized as classical models for HSI classification—utilize a single HSI as input. In contrast, MS2CANet, MFT, MADNet, and PyHypeNet, which are SOTA models for HSI-LiDAR data fusion and classification, incorporate both the HSI and the DSM derived from LiDAR as input modalities.

HybridSN is a hybrid neural network specifically designed for HSI classification. Its core innovation lies in integrating the advantages of 3D convolution and 2D convolution to effectively capture the spatial-spectral joint features in hyperspectral data. SSFTT is based on the Transformer architecture. Through spatial-spectral feature fusion and a self-attention mechanism, it achieves high accuracy and robustness in the HSI classification task. DCTN employs a dual-branch convolutional Transformer architecture and incorporates an efficient interactive adaptive mechanism, thereby achieving outstanding performance in the HSIC task. MS2CANet is a multi-scale pyramid fusion framework that incorporates spatial-spectral cross-modal attention. It enhances the model’s capacity to learn multi-scale information, thereby improving classification accuracy. MFT is a multi-modal fusion transformer network that incorporates the mCrossPA mechanism to integrate complementary information sources with HSI tokens for land cover classification. MADNet is a multi-level attention-based dynamic scaling network that employs an attention module to extract features from HSIs and LiDAR data across multiple levels. PyionNet incorporates a pyramid multi-scale feature extraction module and a progressive cross-fusion mechanism, thereby significantly enhancing the classification accuracy of multi-source data integration.

To ensure the fairness of the experiment, all models are configured with the optimal parameters reported in the literature and are independently executed 10 times using the same training and test sets. Random errors are minimized by statistically analyzing the average performance and standard deviations, thereby improving the comparability across different methods.

#### 4.3.1. Quantitative Analysis

The classification performance of the eight methods was evaluated using three datasets: MUUFL, Trento, and Houston2013. The results of these evaluations are summarized in Table 5, Table 6 and Table 7. For clarity, the highest OA, AA, Kappa coefficient, and per-class classification accuracies are highlighted in bold.

Table 5 presents the classification results of various methods on the MUUFL dataset. The results indicate that the proposed method in this study achieves the highest performance, with an OA, AA, and Kappa coefficient of 88.35%, 87.03%, and 87.43%, respectively. These metrics are 2.27%, 1.46%, and 1.79% higher than those of the second-best method, PyionNet. PyionNet demonstrates relatively balanced classification accuracy across object classes, achieving an OA of 86.08%. In contrast, HybridSN performs the least effectively, with an OA of only 71.5%. Other methods improve feature fusion and spatial-spectral relationship modeling by incorporating attention mechanisms or Transformer architectures, resulting in significantly higher classification accuracy compared to HybridSN. Furthermore, all classification methods that integrate HSI and LiDAR data achieve OA values exceeding 80%, which is notably higher than the performance of the three methods based solely on single-source HSI data.

Table 6 presents a comparison of the classification performance of eight different methods on the Trento dataset. This dataset was acquired from a rural area characterized by large, homogeneous farmlands with significant inter-class spectral variations, thereby offering favorable conditions for classification. The experimental results indicate that, with the exception of HybridSN (OA = 85.51%), the OA of the remaining seven methods exceed 94%. Among them, PyionNet achieved commendable classification performance, with the OA, AA, and Kappa coefficient reaching 98.09%, 97.23%, and 97.65%, respectively. This performance can be attributed to its mechanism of multi-scale feature extraction and cross-fusion. Notably, the method proposed in this study demonstrated superior results, achieving an OA of 99.02%, an AA of 98.59%, and a Kappa value of 99.15%.

Table 7 presents a comparison of the classification performance of eight models on the Houston 2013 dataset. The results indicate that the five methods employing joint classification of HSI and LiDAR data yield significantly better performance than the three methods based on single-source HSI classification. Furthermore, MS2CANet, MADNet, and PyionNet demonstrate superior performance in certain specific categories. For instance, PyionNet achieves the highest classification accuracy for the categories Trees, Water, and Parking Lot2. Notably, the method proposed in this study exhibits outstanding performance, achieving the highest OA (91.85%), AA (92.33%), and Kappa coefficient (91.63%). These results not only validate the effectiveness of multi-modal data fusion in classification tasks but also highlight the superiority of the proposed method in handling complex urban environments.

In conclusion, the following conclusions can be drawn: Across the three datasets, models based on multi-source data demonstrate superior classification performance compared to single-source input models. Among the single-source HSI models, DCTN significantly outperforms SSFT and HybridSN in classification accuracy, as it integrates the technical strengths of both CNNs and Transformer. Among the multimodal models, MADNet employs a spectral angle attention mechanism that enables dynamic scale selection, resulting in overall performance that surpasses that of MS2CANet and MFT, which rely on simple fusion strategies. Furthermore, PyionNet exhibits strong competitiveness due to its efficient fusion architecture. Particularly on the Trento dataset, it achieves an OA of 98.09%, with consistently high and balanced performance across all categories on the other two datasets. Notably, the method proposed in this study achieves the highest classification accuracy across multiple object categories, with an overall accuracy surpassing all comparative models, thereby fully demonstrating its effectiveness and robustness.

#### 4.3.2. Qualitative Analysis

**Figure 8:**
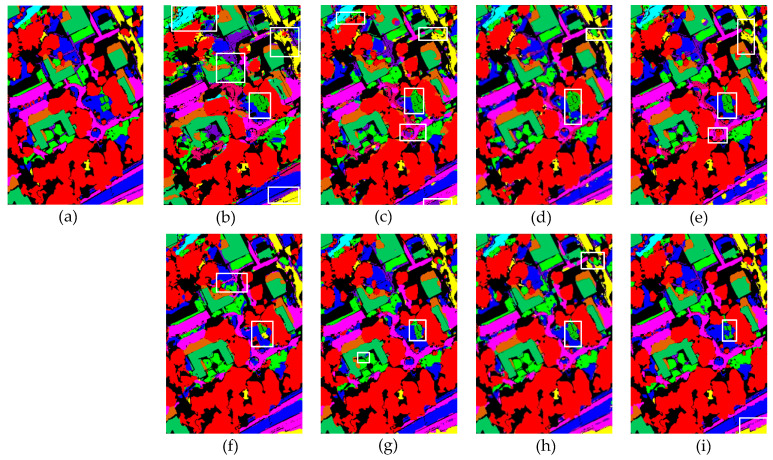
Visualization results of the classification performance of eight different methods on the MUUFL dataset. White wireframes highlight areas with concentrated errors. (**a**) Ground truth. (**b**) HybridSN. (**c**) SSFTT. (**d**) DCTN. (**e**) MFT. (**f**) MS2CANet. (**g**) MADNet. (**h**) PyionNet. (**i**) Proposed method.

**Figure 9:**
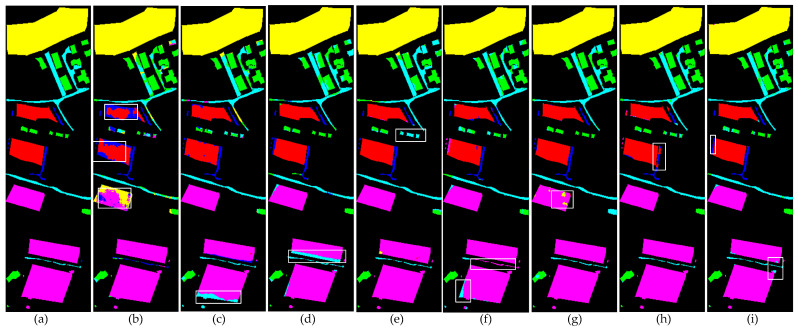
Visualization results of the classification performance of eight different methods on the Trento dataset. White wireframes highlight areas with concentrated errors. (**a**) Ground truth. (**b**) HybridSN. (**c**) SSFTT. (**d**) DCTN. (**e**) MFT. (**f**) MS2CANet. (**g**) MADNet. (**h**) PyionNet. (**i**) Proposed method.

**Figure 10:**
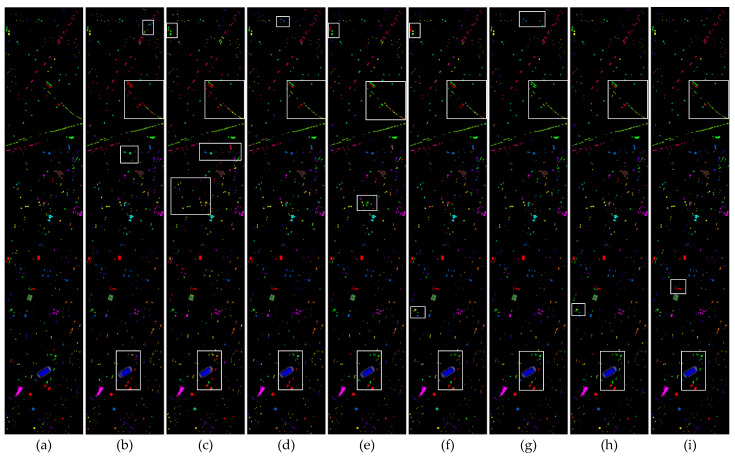
Visualization results of the classification performance of eight different methods on the Houston 2013 dataset. White wireframes highlight areas with concentrated errors. (**a**) Ground truth. (**b**) HybridSN. (**c**) SSFTT. (**d**) DCTN. (**e**) MFT. (**f**) MS2CANet. (**g**) MADNet. (**h**) PyionNet. (**i**) Proposed method.

To systematically evaluate the performance differences between the proposed method and existing comparative approaches, this study conducted qualitative visual analysis experiments on three publicly available datasets. The experimental results are presented in Figure 8, Figure 9 and Figure 10. Through direct comparison of the classification performance of different methods in representative scenarios, the advantages of the proposed method in terms of boundary preservation, detail representation, and noise suppression can be further substantiated.

Figure 8 presents the classification visualization results of each model on the MUUFL dataset. Due to the relatively low spectral discrimination among multiple adjacent object classes in this dataset, single-source input models (HybridSN, SSFTT, DCTN) exhibit noticeable confusion at the boundaries of different land cover types, particularly at the junctions of water/grass/forest and sand/mixed land surfaces in the upper region. Among the multi-source data models, PyionNet demonstrates clear classification boundaries and a competitive visual performance. In comparison, the method proposed in this study excels in detail representation and boundary preservation, effectively mitigating inter-class misclassification and the blurring effect.

Figure 9 presents a comparison of the classification visualization results obtained by various methods on the Trento dataset. As can be observed from the figure, HybridSN produces a significant amount of noise in the vineyard and apple tree regions. SSFTT, DCTN, and MS2CANet exhibit minor misclassifications at the boundary between the lower vineyard and the road. MFT demonstrates noticeable classification errors in the building area. PyionNet shows relatively better performance in these regions, with only a limited number of noisy points appearing in the central ground area. In contrast, the method proposed in this study not only minimizes classification errors but also achieves the best overall classification performance.

Figure 10 presents the visual classification results of eight algorithms on the Houston 2013 dataset. This dataset is characterized by a relatively scattered sample distribution and a complex urban scene. As a result, the first six methods exhibit noticeable classification errors in regions such as railways, stressed grass areas, and the junctions between running tracks and grass in the lower portion of the map. In contrast, PyionNet and the method proposed in this study demonstrate superior classification performance, with no evident misclassification observed.

### 4.4. Ablation Experiment

To evaluate the effectiveness of multimodal fusion in the system, this study designed two experimental frameworks—single-modal (HSI/LiDAR) and dual-modal fusion—for comparative analysis. Experimental results (as presented in Table 8) demonstrate that, across three standard remote sensing datasets, the dual-modal fusion approach substantially outperforms the single-modal approach in various classification metrics. Notably, the performance advantage is more pronounced in test sets characterized by higher scene complexity. This comparative analysis confirms the superiority of the multimodal data fusion strategy in remote sensing image classification, suggesting that the complementary nature of multi-source information can effectively enhance the model’s ability to distinguish complex land cover types.

To systematically evaluate the effectiveness of each module within the model, this study conducted three ablation experiments: (1) EXP1 excluded the shallow multi-scale feature extraction module and retained only the CCformer encoder along with the classifier; (2) EXP2 removed the CCformer encoder and instead employed a basic feature concatenation and fusion strategy in conjunction with the shallow multi-scale feature extraction module; (3) Full denotes the complete model architecture. As presented in Table 9, the experimental results demonstrate that although the CCformer encoder alone (EXP1) achieves relatively high classification accuracy, the integration of the shallow feature extraction module with the CCformer encoder leads to further performance enhancement. Specifically, the full model significantly outperforms the two simplified variants in terms of overall accuracy (OA) and average accuracy (AA), confirming the effectiveness of combining multi-scale feature extraction with the Transformer-based architecture. This comparison clearly illustrates the complementary nature of the model’s components, where the shallow feature extraction module provides a more robust and semantically rich foundation for subsequent high-level feature learning.

## 5. Conclusions

This paper presents a cross-modal cross-attention transformer network designed for the fusion and classification of HSI and LiDAR data. Initially, the proposed method employs a dual-branch shallow multi-scale feature extraction module to separately capture the spectral-spatial features of HSI and the elevation features from LiDAR data. Subsequently, a cross-attention mechanism is integrated into the Transformer architecture to jointly guide and fuse the early-stage features of both modalities, thereby enabling adaptive feature complementation and enhancement across modalities. Experimental results on three widely recognized datasets—MUUFL, Trento, and Houston2013—demonstrate that OA achieved by the proposed method reaches 88.35%, 99.02%, and 91.85%, respectively. The method significantly outperforms existing state-of-the-art approaches, thereby validating its effectiveness and technical advancement. This study offers a novel approach for multi-modal remote sensing data fusion and classification. In future work, we plan to investigate self-supervised pre-training strategies for multi-modal feature learning to enhance generalization in unsupervised scenarios.

## Figures and Tables

**Table 1:** Summary of HSI datasets.

	MUUFL	Trento	Houston2013
Data Type	HSI, DSM	HSI, DSM	HSI, DSM
Samples	53,687	30,214	15,029
Number of Classes	11	6	15
pixels	325 × 220	166 × 600	349 × 1905
Resolution (m)	0.54 × 1.0	1	2.5
Band Number	64 (HIS)	63 (HSI)	144 (HSI)
Area and Country	Mississippi, USA	Italy	Texas, USA

**Table 2:** The labels and quantities of the samples in the MUUFL dataset.

Class	Category	Train Samples	Test Samples	Total Samples
01	Trees	20	23,226	23,246
02	Mostly Grass	20	4250	4270
03	Mixed Ground Surface	20	6862	6882
04	Dirt and Sand	20	1806	1826
05	Road	20	6667	6687
06	Water	20	446	466
07	Building Shadow	20	2213	2233
08	Building	20	6220	6240
09	Sidewalk	20	1365	1385
10	Yellow Curb	20	163	183
11	Cloth panels	20	249	269
	Total Samples	220	53,467	53,687

**Table 3:** The labels and quantities of the samples in the Trento dataset.

Class	Category	Train Samples	Test Samples	Total Samples
01	Apple Trees	20	4014	4034
02	Building	20	2883	2903
03	Ground	20	459	479
04	Woods	20	9103	9123
05	Vineyard	20	10,481	10,501
06	Roads	20	3154	3174
	Total Samples	120	30,094	30,214

**Table 4:** The labels and quantities of the samples in the Houston2013 dataset.

Class	Category	Train Samples	Test Samples	Total Samples
01	Health Grass	20	1231	1251
02	Stressed Grass	20	1234	1254
03	Synthetic Grass	20	677	697
04	Trees	20	1224	1244
05	Soil	20	1222	1242
06	Water	20	305	325
07	Residential	20	1248	1268
08	Commercial	20	1224	1244
09	Road	20	1232	1252
10	Highway	20	1207	1227
11	Railway	20	1215	1235
12	Parking Lot1	20	1213	1233
13	Parking Lot2	20	449	469
14	Tennis Court	20	408	428
15	Running Track	20	640	660
	Total Samples	300	14,729	15,029

**Table 5:** The quantitative comparison results of 8 methods on the MUUFL dataset.

Class No.	HSI Input			HSI and LiDAR-DSM Input				
	HybridSN	SSFTT	DCTN	MFT	MS2CANet	MADNet	PyionNet	Proposed
1	88.44	88.89	84.73	82.51	83.12	**90.17**	87.46	90.15
2	71.26	72.85	74.61	70.62	74.33	80.36	80.11	**83.97**
3	49.78	62.81	67.47	68.46	81.53	69.94	80.80	**85.03**
4	73.20	**90.86**	78.17	75.67	80.66	89.80	87.51	89.20
5	34.80	63.85	68.44	77.35	82.56	88.09	83.40	**93.21**
6	95.51	97.33	94.21	97.15	96.99	**98.90**	95.32	95.71
7	62.00	83.33	75.55	80.10	83.12	86.96	82.58	**87.93**
8	84.22	78.99	88.36	90.33	89.88	90.08	88.33	**91.51**
9	40.90	43.59	66.19	69.69	74.68	70.28	**76.90**	75.73
10	60.11	77.91	79.77	71.36	83.37	**89.28**	88.91	85.44
11	90.47	89.56	**91.09**	90.79	89.44	90.46	89.99	87.38
OA (%)	71.50 ± 2.51	78.77 ± 1.77	80.11 ± 1.89	81.46 ± 1.99	82.98 ± 1.77	85.65 ± 1.77	86.08 ± 1.88	**88.35 ± 1.87**
AA (%)	68.45 ± 3.08	77.45 ± 2.33	80.71 ± 2.62	81.10 ± 2.70	83.61 ± 1.60	85.85 ± 1.24	85.57 ± 2.01	**87.03 ± 1.13**
Kappa × 100	65.96 ± 3.05	72.64 ± 2.59	81.20 ± 3.21	81.00 ± 2.89	82.90 ± 1.86	86.10 ± 1.38	85.64 ± 1.98	**87.43 ± 1.51**

The bolded value indicates the optimal value.

**Table 6:** The quantitative comparison results of 8 methods on the Trento dataset.

Class No.	HSI Input			HSI and LiDAR-DSM Input				
	HybridSN	SSFTT	DCTN	MFT	MS2CANet	MADNet	PyionNet	Proposed
1	64.36	97.79	95.88	95.09	98.18	98.00	95.47	**99.37**
2	83.21	77.26	87.68	**99.07**	95.54	97.09	97.07	97.68
3	95.45	96.59	98.67	89.84	95.51	96.39	**100**	97.36
4	97.71	99.95	99.76	98.08	**100**	99.99	98.11	**100**
5	89.09	98.99	95.80	98.16	97.50	98.99	98.07	**99.95**
6	67.23	89.97	90.21	89.99	91.40	90.77	94.65	**97.20**
OA (%)	85.51 ± 2.61	94.11 ± 1.87	95.41 ± 2.19	95.01 ± 1.97	96.35 ± 1.88	96.62 ± 1.75	98.09 ± 1.17	**99.02** ± 1.39
AA (%)	82.85 ± 3.08	93.67 ± 2.14	94.89 ± 1.94	95.10 ± 2.22	97.36 ± 1.59	96.87 ± 2.49	97.23 ± 0.70	**98.59** ± 1.50
Kappa × 100	82.11 ± 2.93	94.07 ± 2.19	94.20 ± 1.75	95.44 ± 1.69	96.03 ± 1.71	97.09 ± 1.98	97.65 ± 1.25	**99.15 ± 1.37**

The bolded value indicates the optimal value.

**Table 7:** The quantitative comparison results of 8 methods on the Houston2013 dataset.

Class No.	HSI Input			HSI and LiDAR-DSM Input				
	HybridSN	SSFTT	DCTN	MFT	MS2CANet	MADNet	PyionNet	Proposed
1	48.23	**91.78**	80.55	76.67	80.39	77.59	89.31	83.67
2	84.68	78.42	91.23	93.02	91.47	**98.90**	93.22	90.78
3	75.47	80.57	92.34	95.44	**99.89**	90.55	99.68	98.90
4	69.28	90.98	89.16	95.33	97.56	95.15	**96.11**	96.00
5	85.14	87.07	92.11	94.60	**98.57**	93.66	95.47	94.67
6	87.39	90.92	91.58	93.47	98.01	94.22	**98.25**	98.06
7	65.79	93.02	90.26	86.29	80.73	89.61	84.12	**94.34**
8	91.33	92.36	87.60	84.39	90.21	89.69	87.95	**94.23**
9	71.87	93.02	86.16	84.77	85.71	**95.33**	75.66	90.56
10	86.72	82.60	79.12	65.99	67.23	80.81	89.17	84.34
11	88.90	77.68	88.09	92.19	94.16	**94.99**	90.21	90.69
12	69.02	70.36	87.34	87.48	**93.19**	88.96	90.68	93.06
13	80.14	91.82	91.56	84.99	78.87	85.86	**94.73**	94.33
14	90.70	91.71	92.40	93.18	90.19	94.76	96.65	**97.30**
15	65.77	89.87	85.23	**96.77**	90.25	94.22	93.21	90.11
OA (%)	74.32 ± 1.51	83.05 ± 1.77	87.99 ± 2.27	88.12 ± 2.07	88.13 ± 1.78	89.60 ± 1.95	90.01 ± 1.27	**91.85 ± 1.15**
AA (%)	75.24 ± 1.48	84.40 ± 2.84	88.29 ± 2.08	88.30 ± 2.48	89.10 ± 1.85	90.96 ± 2.20	91.21 ± 1.33	**92.33 ± 0.93**
Kappa × 100	66.89 ± 1.25	81.79 ± 1.49	88.20 ± 1.88	88.07 ± 1.88	88.02 ± 1.77	90.11 ± 1.59	90.25 ± 1.78	**91.63 ± 1.28**

The bolded value indicates the optimal value.

**Table 8:** Experimental Comparison Results under Varying Input Conditions.

Input Case	MUUFL			Trento			Houston2013		
	OA (%)	AA (%)	K × 100	OA (%)	AA (%)	K × 100	OA (%)	AA (%)	K × 100
HSI	81.38	80.70	80.98	93.56	94.25	92.55	85.58	86.29	85.64
LiDAR	57.47	55.76	54.22	64.77	65.32	63.16	69.98	71.43	68.86
HSI + LiDAR	**88.35**	**87.03**	**87.43**	**99.02**	**98.59**	**99.15**	**91.85**	**92.33**	**91.63**

The bolded value indicates the optimal value.

**Table 9:** Comparative Results of Ablation Experiments.

Exp	MUUFL			Trento			Houston2013		
	OA (%)	AA (%)	K × 100	OA (%)	AA (%)	K × 100	OA (%)	AA (%)	K × 100
Exp1	79.44	79.56	78.87	87.86	88.98	87.84	82.18	83.36	81.47
Exp2	49.35	50.85	48.95	55.33	58.47	56.66	51.66	51.69	50.11
Full	**88.35**	**87.03**	**87.43**	**99.02**	**98.59**	**99.15**	**91.85**	**92.33**	**91.63**

The bolded value indicates the optimal value.

## Data Availability

Data are contained within the article.

## Abbreviations

The following abbreviations are used in this manuscript:

HSI	Hyperspectral image
HSIC	Hyperspectral image Classification
LiDAR	Light Detection and Ranging
CNN	Convolutional Neural Network
SVM	Support Vector Machine
ELM	Learning Machine
RF	Random Forest
ViT	Vision Transformer
FPN	Feature Pyramid Network
RNN	Recurrent Neural Network
NLP	Natural Language Processing
DSM	Digital Surface Model
OA	Overall Accuracy
AA	Average Accuracy
